# Using online technologies to improve diversity and inclusion in cognitive interviews with young people

**DOI:** 10.1186/s12874-020-01024-9

**Published:** 2020-06-16

**Authors:** Ushma D. Upadhyay, Heather Lipkovich

**Affiliations:** 1 Department of Obstetrics, Gynecology and Reproductive Sciences, Advancing New Standards in Reproductive Health (ANSIRH), University of California, San Francisco, 1330 Broadway, Suite 1100, Oakland, CA 94612 USA; 2 University of California Global Health Institute, Center of Expertise on Women’s Health, Gender, and Empowerment, San Francisco, California USA; 3 Department of Surgery, University of Michigan, 2800 Plymouth Road, Ann Arbor, MI 48109 USA

**Keywords:** Adolescents, Youth, Young adults, Cognitive interviews, Qualitative research, Online research methods, Recruitment, Video interviewing, Social distancing, Diversity, Inclusion, Race/ethnicity

## Abstract

**Background:**

We aimed to assess the feasibility of using multiple technologies to recruit and conduct cognitive interviews among young people across the United States to test items measuring sexual and reproductive empowerment. We sought to understand whether these methods could achieve a diverse sample of participants. With more researchers turning to approaches that maintain social distancing in the context of COVID-19, it has become more pressing to refine these remote research methods.

**Methods:**

We used several online sites to recruit for and conduct cognitive testing of survey items. To recruit potential participants we advertised the study on the free online bulletin board, Craigslist, and the free online social network, Reddit. Interested participants completed an online Qualtrics screening form. To maximize diversity, we purposefully selected individuals to invite for participation. We used the video meeting platform, Zoom, to conduct the cognitive interviews. The interviewer opened a document with the items to be tested, shared the screen with the participant, and gave them control of the mouse and keyboard. After the participant self-administered the survey, the interviewer asked about interpretation and comprehension. After completion of the interviews we sent participants a follow-up survey about their impressions of the research methods and technologies used. We describe the processes, the advantages and disadvantages, and offer recommendations for researchers.

**Results:**

We recruited and interviewed 30 young people from a range of regions, gender identities, sexual orientations, ages, education, and experiences with sexual activity. These methods allowed us to recruit a purposefully selected diverse sample in terms of race/ethnicity and region. It also may have offered potential participants a feeling of safety and anonymity leading to greater participation from gay, lesbian, and transgender people who would not have agreed to participate in-person. Conducting the interviews using video chat may also have facilitated the inclusion of individuals who would not volunteer for in-person meetings. Disadvantages of video interviewing included participant challenges to finding a private space for the interview and problems with electronic devices.

**Conclusions:**

Online technologies can be used to achieve a diverse sample of research participants, contributing to research findings that better respond to young people’s unique identities and situations.

## Background

Cognitive interviewing to test survey items is a vital step in psychometric scale development [1, 2]. It ensures that it is not too difficult to access the needed information from memory and that items are worded clearly and at a level that participants can understand [3]. Cognitive interviewing among adolescents and young adults is particularly important because there is substantial variation in developmental stages, cognitive abilities, and vocabulary levels in these age groups [4]. Additionally, for psychometric instruments related to sexual and reproductive health for young people, a wide variety of nomenclature and slang is used to describe sexual activity among different sub-populations and friend groups [5–7]. Words that are used among members of one group may not be used by members of another or they may have a different meaning. Additionally, lesbian, gay, bisexual, and trans people may have unique perspectives related to sexual and reproductive issues [8]. Cognitive interviewing helps determine whether the scale’s items and terminology are being interpreted as intended across diverse sociodemographic backgrounds [9].

Too often, however, researchers use convenience sampling methods for cognitive interviewing without attention to the sociodemographic characteristics of the participants [10]. Adolescent participants are often selected from existing health programs [11], schools, or clinics [12, 13]. Consequently, researchers identify only those issues identified as problematic for adolescents with exposure to health or school structures. This may result in question items that, while acceptable to majority groups, unintentionally marginalize minority groups. For example, researchers recruiting for cognitive testing at a general pediatric clinic may never assess how their questions fare among transgender populations. Thus, their final survey items may only apply to cis-gender young people.

Few previous studies have focused on the importance of cognitive interviewing among diverse samples when developing new measures [9]. In one study, analysis of cognitive interviews identified problems in many of the 159 items because items were not interpreted the same across all racial/ethnic groups [14]. More common, however, are critiques that a particular measure, after it has already been developed, is not as valid or useful among a specific population [8].

Online message boards, email, and video technologies provide an opportunity for researchers to both recruit and conduct interviews with participants who may otherwise be underrepresented in cognitive interviewing. Of teens (ages 13–17), 97% report going online, with 45% saying they are online constantly [15]. This near ubiquity of internet access provides an opportunity for researchers to recruit outside of traditional brick and mortar spaces.

Understanding the role of remote technologies for conducting interviews has become all the more relevant in response to the increased use of telehealth and the emerging need for remote research methods that maintain social distancing in the context of COVID-19. Compared to in-person methods, using remote technologies to conduct the interviews may present opportunities for expanding inclusivity. Research comparing video to in-person interviews for qualitative research cite reduced expense and time consumed by travel to different locales, easier scheduling, and reduced costs in terms of staff time [16–18]. These advantages may enable low-income, less mobile, and rural individuals to participate in research [19]. Additionally, the anonymity of video interviewing may be an added benefit. In fact, one study on men who pay for sex found that video interviewing facilitated recruitment of this hard-to-reach group because of the research participants’ profound desire to protect their identities [20].

In this study we aimed to assess the feasibility of using multiple technologies to recruit and conduct interviews with a population of young people about sexual and reproductive health and empowerment. We also aimed to understand whether these methods could contribute to a diversified sample of cognitive interview participants. We describe our methods, providing sufficient detail and recommendations for improving our methods, to guide researchers who wish to use online technologies in their research.

## Methods

This study describes the initial steps of a larger project to develop a validated measure of young people’s sexual and reproductive empowerment [21]. Between May 2017 and February 2018 we conducted cognitive interviews to pretest an item pool of 120+ items.

### Ethics, consent and permissions

The methods were approved by the institutional review board (IRB) of the University of California, San Francisco (UCSF) (Approval # 16–21267). The IRB required the participant’s consent but did not require parental consent or notification for minors because in research on sexual and reproductive health topics, which are highly stigmatized for adolescents, attempting to obtain parental consent could compromise the adolescents’ guarantee of confidentiality, thus potentially resulting in additional risks to the minor. Additionally, actual responses to the scale items were not the primary interest of the cognitive interviews. All modifications to our procedures described below also received IRB approval. Participants received a $25 electronic gift card within 24 h of participation.

We recruited for the study on the Community/Volunteers section of Craigslist in selected cities/states and the Reddit subreddit r/samplesize. We posted individual ads on each entitled: “Participants (15–24) needed for interview research study.” We subsequently added “Get $25!” which resulted in an increase in responses. We chose these two websites because they were free to post to for researchers and free for potential participants to access, and both sites allowed ads for research participation. In addition we chose them based on our knowledge that these sites are popular among young people (particularly Reddit).

Ads were posted from May 2017 to January 2018. Respondents clicked on a link that opened a Qualtrics screening survey asking their age, race/ethnicity, level of education, and status as a current student. Eligibility was limited to those ages 15–24 who also indicated on the survey that they could meet either online or in a public place in the Atlanta area (where the Principal Investigator was based at the time) and were willing and able to participate in the cognitive interview session. Qualtrics is a secure, HIPAA compliant survey research platform. While the survey existed on the UCSF Qualtrics platform, only members of the study team had access to this survey and the associated responses. Excel files generated by Qualtrics were downloaded from the platform directly to a password protected study folder on the UCSF server. These files were also kept on a secure Box folder available only to the study team. Identifiable information provided in the Qualtrics survey was not linked to cognitive interview responses. All files with identifiable information were only accessed behind the firewall of the team’s respective institutions.

We initially posted only on Craigslist of Atlanta to allow these participants to decide whether to meet online or in a neutral, public space. Because so few participants opted to meet in person, and when they did at least two did not come at the agreed upon time and place, we subsequently removed the in-person option. We expanded the online interviews by listing on more Craigslist sites of large and medium sized US cities that represented all geographic regions of the US. We then began posting on Reddit and participants from anywhere in the United States could respond to the screening survey.

All communications related to planning for the interview were done over email. In the initial phases, the study coordinator emailed all eligible respondents ages 15 to 24 who completed the screening survey. As certain age, education, gender, and racial/ethnic groups reached saturation based on the study target sample goals, the coordinator then prioritized email invitations to those whose demographics were underrepresented in the completed interviews. Eligible respondents in these prioritized groups were invited to participate in an interview via the email address they provided. The research coordinator and the researcher used their UCSF email addresses, not a study email account and each email was personalized. The subject of the email stated that this was an invitation to participate in a “UCSF Study Feedback Interview” and the body of the email contained an introduction to the study, the name of the researcher, details on confidentiality, a list of available interview times in the respondent’s time zone, and the details of remuneration for participation. We did not send any follow-up emails to those who did not respond. As recruitment slowed down, the subject line of this email was amended to include that a $25 gift card would be provided for participation. Interested respondents replied to the email, confirming their interest and preferred interview time. After an interview time was agreed upon, the coordinator emailed additional information including further details about the interview and interviewer (including a link to her university webpage with biography and headshot), instructions for accessing the video meeting app, a request that they use a computer if possible for the meeting for ease in reading and marking up the survey, and information on how to receive their gift card after the interview.

The coordinator and/or the researcher continued email correspondence with those participants who had further questions about the interview logistics or who needed to reschedule. Participants who missed their initial interview date were followed up with at least once to offer a chance to reschedule their interview at a more convenient time.

To conduct the interviews we used Zoom Enterprise Video Communications (www.zoom.us, San Diego, CA), which is available for use at no cost to UCSF researchers. At the prearranged date and time, the participant and researcher both clicked on the Zoom link and entered the private video chat room. The cognitive interviews involved the researcher first sharing her screen with the participant and reviewing an informed consent form together, reading aloud major portions and ensuring participant comprehension. The researcher asked participants their age and minors received additional information on mandated reporting. Additionally, the researcher assured the participant that there was no one in her room and that because she was wearing headphones, no one could hear what they said. After verbal consent to participate was given, all participants were offered an email copy of the consent form. Next, the researcher asked participants to tell her about themselves, to obtain a better understanding of how their unique situation may affect their comprehension or interpretation of the draft scale items. The researcher then opened the Microsoft Word document with the item pool, shared her screen, gave control of the mouse and keyboard to the participant, read aloud the instructions and asked them to self-administer one part of the survey, selecting the best response choices. After the participant completed all items in the section, the researcher probed about interpretation and comprehension, asking for paraphrasing of the questions, and general probing such as, “How did you arrive at that answer?” and “Given your specific situation, did you feel that question applied to you?” Most follow-up questions resembled qualitative interview probes, such as, “Tell me about the circumstance in your life that made you respond in that way.” or “What did you think about that made you select that response?” After one section was complete they moved to another section. The interviews were not recorded but the researcher took notes on the responses.

After all of the interviews were completed, we wanted to learn more about the participants’ experiences. In March 2018, with IRB approval, we sent all 30 participants an email inviting their participation in a follow up survey. The email stated we were inviting them to take a “5 minute, online follow-up survey to help us and other researchers learn about better ways to conduct interviews like the one you did.” The email contained a link to a Qualtrics survey which asked about 20 additional general questions about their impressions of the research methods, particularly as they related to the technology used. Participants were asked several questions on how easy or difficult it was to schedule the interview, how easy or difficult it was to do the interview over video chat, how convenient or inconvenient it was, how comfortable or uncomfortable they were speaking about their personal life with the researcher over video chat, and how private or public it felt. Finally we asked their preferred method of being interviewed. The survey was open for 2 weeks. An email was sent to each participant who completed the follow-up survey with a link to a $10 gift card in remuneration for their time.

## Results

A total of 672 completed our screening survey and we purposively selected 117 to participate in the cognitive interviews. In total, we recruited and interviewed 30 participants. All participants were recruited using online methods, and all interviews, except one, were conducted using a virtual platform. We were able to purposively sample a diverse group of young people. We obtained a sample of young people of a variety of ages, and the majority of respondents were people of color. Most participants were currently students, reflective of the young age, and the largest group of participants came from the South while none were from the West in the United States (see Table 1). Of the total sample, 27 completed the follow-up survey. All respondents to the follow-up survey had conducted their interviews with the researcher virtually. Of the three non-respondents, 2 were from Atlanta, 2 were male identified, and 2 identified as black. Two of the individuals were in their 20’s while the other was in their early teens.

**Table 1:** Sociodemographic characteristics of the interview participants (*n* = 30)

	Number	% of Total^a^
Age		
15–17	8	27%
18–20	10	33%
21 – 24^b^	12	40%
Race		
White	7	23%
Black	16	53%
Asian	5	17%
American Indian or Alaska Native	1	3%
Multiracial	1	3%
Ethnicity		
Hispanic/Latinx	3	10%
Non-Hispanic/Non-Latinx	26	87%
Unknown	1	3%
Gender Identity		
Cisgender Woman	18	60%
Cisgender Man	11	37%
Transgender	1	3%
Sexual Orientation		
Gay or Lesbian	2	7%
Bisexual or Bi-Curious	1	3%
Heterosexual	27	90%
Highest Level of Education Attained		
Some High School^c^	8	27%
High School Graduate	5	17%
Associates Degree or some other college or technical school	12	40%
College Graduate or Postgraduate	5	17%
Currently a student		
Yes	23	77%
No	7	23%
Region		
Northeast	8	27%
Midwest	8	27%
South	14	47%
Raised by Birth Parent(s)	26	87%
Raised by Adoptive Parent(s)	1	3%
Raised by a Guardian (foster parent or relatives)	3	10%

^a^Percentages may not add to 100 due to rounding

^b^One participant stated on the eligibility survey that they were 24. During the interview they disclosed an age of 26

^c^“Some high school” captures those still in high school or who have stopped attending high school but did not graduate

We first present our findings related to online recruitment and then describe findings related to conducting interviews using video chat.

### Online recruitment

Using online platforms to advertise the study and then screen participants allowed us to purposefully recruit a more diverse sample in terms of race/ethnicity and region. Online recruitment also offered potential participants a feeling of anonymity that may have fostered greater participation or disclosure from stigmatized or marginalized groups or shy individuals that would not have otherwise agreed to participate. These themes are explained with more detail below.

#### Diversity in race/ethnicity

We included items on race and ethnicity in the screening survey and then purposively sampled for participants who selected non-white races or ethnicities. This process led to a diverse sample including 16 who identified as black or African American, 5 as Asian, 1 as American Indian, and 1 as multiracial. Among the 30, 3 identified as Hispanic or Latinx.

Diversity in race/ethnicity also meant that the sample included international representation. One participant from Turkey discussed cultural differences in gender norms that had implications for some of the items. Likewise, comments from another participant of Indian ethnicity who was raised by relatives in the U.S. after his parents returned to their home country, led to changes on items related to parental involvement that would not have otherwise been made.

#### Diversity in gender identity, sexual orientation, and sexual experience

Our recruitment methods were not intentionally designed to maximize diversity in gender identity or sexual orientation, for example, we did not ask about gender identity or sexual orientation on the screening form, but using online platforms may have afforded participants in more marginalized groups a feeling of safety in volunteering to participate. Individuals were likely more comfortable volunteering without fear of being persecuted. Among the 30 participants, we recruited one person who identified as a gay man, one person who identified as a “bi-curious” man, one woman who reported currently having a girlfriend, and one person who identified as a transgender man. Having some representation from sexual minority groups in cognitive testing for a scale on sexual health and reproductive empowerment is vital due to the stigma and discrimination they face. Additionally, it allowed us to test a question on condom use with a woman who has sex with women. Screening and purposefully selecting on these identities or characteristics would have resulted in even greater representation.

Additionally, the online recruitment methods allowed greater variation in sexual experience than we would have found had we recruited at a sexual health or family planning clinic where patients are mostly those who have been sexually active. This was important as we wanted to ensure that the items were comprehensible and applicable to youth who had never had sex (regardless of sexual orientation). While we did not screen for or select participants based on sexual experience, we interviewed 9 participants who had never had sex. Their responses led to edits to the wording of a few questions to expand applicability. For example, the item, “I worry that a romantic partner could interfere with me achieving my life goals” was changed to “I worry that *someday* a romantic partner could interfere with me achieving my life goals.” The revised item makes it more applicable to an adolescent who is not currently in a romantic relationship as well as removes the emphasis from the current partner for those who are currently in a relationship. Based on a participant’s suggestion we added to the instructions: “You don’t need to have ever had sex or currently have a sexual partner to answer this survey. If you are unsure about how to answer any items, please give your best guess.”

#### Regional diversity

Systematically and intentionally posting in a variety of regions and selecting participants based on region of residence led to greater diversity in our sample. Large urban areas in the United States tend to have more people of color, people of marginalized identities, educated populations, and more liberal viewpoints about sexuality and sex education than mid-size and smaller cities [22]. Including large cities as well as smaller cities in our recruitment allowed us to achieve diversity in terms of gender identity, race/ethnicity, and education, while still including participants from rural areas. We varied Craigslist postings in cities and regions based on the website’s rules and posting position (i.e. a posting in Kansas City may have stayed on the first page for 45 days but a posting in Atlanta was knocked off the first page by day 2 of its posting life). Additionally, posting frequency was not necessarily related to the number of responses to the posting. For example, we posted 5 times to 10 cities in the Northeast and 28 times to 10 cities in the Midwest yet had 8 participants from each of those regions. Although we posted in 11 cities in the West for a total of 14 postings, responses from the Mountain and Western regions were sparse. This may be because while on faculty at UCSF, the researcher was based in Atlanta, Georgia during the research period. Scheduling a time for West Coast young people interested in participating proved to be a challenge. For our research, the researcher’s time constraints and inability to regularly schedule evening interviews was a limitation that prolonged the recruitment period.

### Conducting video interviews

Conducting the interviews using video chat offered several advantages including offering safety and anonymity for hard-to- reach young people, allowing for appropriate levels of parental involvement, flexibility in scheduling, fostering feelings of safety, and completion of the instrument electronically. Disadvantages included participant challenges in finding a private space for the interview and challenges related to participants’ electronic devices. Below we expand on these themes.

#### Engaging hard-to-reach youth

The prospect of engaging in research over video-chat may have facilitated participation from certain individuals who otherwise would not have volunteered for research if recruited at healthcare centers or afterschool programs using in-person methods. For example one participant grew up in foster homes, never knew his parents, and never had any adult role models in his life. Feedback from this participant was useful in assessing items referring to parents/guardians. He deemed the item, “Talking about my problems with my parents/guardians makes me feel ashamed or foolish” not applicable to him because it assumes the participant has a relationship with a parent or guardian. We subsequently deleted the item from our item pool.

We also interviewed participants who were shy, introverted or otherwise reluctant to engage in an in-person conversation. One participant reported that she does a lot with technology including an internship at her school and is always at her computer. She identified as shy and throughout the interview had a piece of cloth over the camera to obscure her identity. Additionally, women and transgender people generally may be unwilling to meet strangers even in public for in-person interviews due to safety concerns. When asked if she would have met the researcher at a café, one female participant flatly said “No I wouldn’t have. I can stop this at any time. I am in my safe zone here and if this went bad I could disconnect it.”

#### Scheduling

Among the 27 participants who completed the follow-up survey, 23 reported that it was “easy” or “very easy” to schedule interviews over emails (see Table 2). In follow-up, participants commented that email scheduling was easy for reasons including “all the info I needed was right there in the email,” that email “helps avoid miscommunications” and “it gave me a chance to look over my schedule [ …] and not feel pressured like I would over the phone.” Interviews were scheduled based on respondents’ schedules, convenience, and when they would have privacy. Offering such flexibility made it easier to participate, which may have helped reduce volunteer bias for those with demanding schedules. Due to work and school schedules, young people who are low-income may not have a lot of free time to participate in research, even if compensated. One challenge, however, was trying to find mutually convenient times when there were large differences in time zones between the researcher and potential participant, some of which was negated by offering flexibility with the time and day of interview, including sometimes offering evening and weekend appointments.

**Table 2:** Post-Interview Responses to Opinions About Using Technology for Interview (*n* = 27)

Response	Number	% of Total^a^
Reported easy or very easy to schedule video interview over emails	23	85%
Preferred method of scheduling interview		
Email	17	63%
Phone call	5	17%
Text	5	17%
Other	0	0%
Reported easy or very easy to use video chat for interview	18	67%
Reported convenient or very convenient to use video chat for interview	24	89%
Reported comfortable or very comfortable speaking about personal life with the researcher over video chat	19	70%
Reported private or very private to use video chat for interview	22	81%
Device used for interview		
Smartphone	4	15%
Tablet	3	11%
Computer	20	74%
Preferred method for interview		
Video chat – Zoom or other	23	85%
In person	2	7%
Other place/method	1	4%
No Response	1	4%

^a^ Frequencies may not add to 100 due to rounding

In the follow-up survey, 24 participants reported that being interviewed over video chat was “convenient” or “very convenient.” Several stated that they preferred video meeting because they did not have to leave the house, saving them time, money, and/or anxiety. As one participant explained, “If I had to go to an interview at a certain place I feel like I would get lost or show up late, but with Zoom its right on my computer.” When asked whether he would have agreed to participate if we met at a local café one participant said we would have to compensate him a lot more: “I did this because it was really easy.”

#### Rapport

Most participants reported that meeting by video gave them a sense of safety and security that enabled them to open up with the interviewer. Technology may have helped build the participant’s trust in the researcher. Because all participants received a link to the researcher’s university webpage, they could view the researcher’s credentials and image before the interview and then visually verify the researcher’s identity during the video chat. They were able to see in advance that the researcher was a woman of color, and for some potential participants of color, that may have increased feelings of safety, comfort and willingness to participate.

In the follow up survey, 18 out of 27 reported it was “easy” or “very easy” to do the interview over video and 19 reported it was “comfortable” or “very comfortable” speaking about their personal life with the researcher over video chat (see Table 2). Many spoke from home or school in a private location where they felt their confidentiality could be maintained. One said, “I got to choose the time and location at which I spoke, putting me in control of who was able to see and hear me.” Another participant said, “I think a more public chat would be weird because I would feel pressured but instead it was more private, and I could be interviewed knowing it’s just me and the person talking alone not being watched by others.” According to one participant who indicated in follow-up that they would choose a video interview over other modalities, video chat “takes out transportation costs, feels safer, enhances the volunteer aspect of the interview since I felt more in control with regards to how and when I could terminate the interview if I felt uncomfortable.”

Nevertheless, a few respondents struggled to find a private place for a video chat. Two participants did the interview in an empty school classroom and one did it from a café that did not afford her sufficient privacy. Midway through the interview she moved to a bench outside the café.

Additionally, 5 of the participants reported it was “public or very public” and at least 2 of the 27 follow-up participants reported not feeling safe in a video interview. One reported not wanting to open up to a stranger on video during the interview. She mentioned a few tough life experiences that she didn’t “want to get into.” When asked about the video interview method she said she found it difficult to share the intimate details of her life over video. She would prefer to meet in person and said she would definitely have met the researcher at a café had that opportunity been available.

#### Technical issues

For the researcher, advantages of a video interview included the ability to verify the participants’ age visually and the ability to share their screen and remote control while the participant completed the survey. This enabled the participant to complete the survey on the shared screen and the researcher to monitor the pace at which the participant completed survey items and note items that took longer to respond to.

Among the 27 participants who completed the follow up survey, 9 reported that using the technology was difficult. These technical difficulties included having trouble downloading the Zoom app, locating a computer with a camera for the video chat, not being able to get audio, and taking the “remote control” (which allowed the respondent to select responses in real time). We preferred that respondents use a computer because the draft items were iteratively updated in Microsoft Word, and not optimized for smartphone use. When participants did not have a computer, they connected via smartphone, which seemed technologically easier, however they were then unable to self-administer the survey. In these cases they read aloud their responses and the researcher marked their survey responses. For the audio issues, simultaneous phone calling helped reduce problems for some.

#### Parental involvement

While we did not require parental consent, a decision approved by our IRB, using video meeting could facilitate parental supervision when desired by the participant. On two occasions, participants’ mothers accompanied the participant for the first few minutes of the interview. They met the researcher to verify the authenticity of the meeting and on one occasion assisted the participant to locate headphones to improve audio quality. Parental attendance during the interview would have been permitted but it might have affected respondents’ ability to discuss their lives candidly. A recent meta-analysis found that requiring parental consent in research can lead to a systematic bias in the sample where the population of minors under study is underrepresented [23]**.** Online video meeting may have facilitated adolescent participation without obtaining parental permission. As one participant stated, “Zoom was the most suitable place because of convenience, I am not likely to get around due to strict parents.”

## Discussion

These findings confirm that online recruitment and interviewing methods are feasible and useful for obtaining a diverse set of cognitive interviews. These results are consistent with previous research concluding that online recruitment can lead to diverse samples [24] and that video interviewing increases convenience [16–18], and feelings of safety [25], facilitating inclusion of hard-to-reach or stigmatized groups. Additionally, given the comfort that most participants reported feeling, the quality of responses gained through online research is likely to be similar to responses produced by more traditional methods of cognitive interviewing.

This study demonstrates that online recruiting is promising for cognitive interviewing when used with purposive sampling based on sociodemographic characteristics. Previous studies using online recruitment describe using Google and Facebook ads for recruiting large samples for quantitative studies [26, 27]. Such studies introduce concerns that online recruitment may lead to biased sampling, for example, overrepresentation of more educated people [28, 29]. Because cognitive interviewing aims to recruit diverse samples, and not necessarily a representative sample of participants, online recruitment is particularly well-suited to cognitive interviewing.

This study has implications for qualitative interviewing as well. These online recruitment and interviewing methods could be employed for research involving individual in-depth interviews. Qualitative research is enriched when samples are diverse and represent a variety of experiences [30, 31]. Our methods involved using many probes akin to those used in qualitative research; thus participants may feel the same safety and comfort with in-depth interviews over video.

When considering recruitment and interviewing methods, researchers must consider financial costs. Posting on Craigslist and Reddit was free and there are several online video chat apps that are available free of charge. Nevertheless, recruiting online requires substantial amounts of person time to manage the posting of advertisements following appropriate site guidelines, ensuring that postings are sufficiently spaced and monitored for spam “flagging” in order to confirm that advertisements are reaching their intended audience. These requirements necessitate substantial time from study staff. Emailing and scheduling participants is also time consuming. We had to invite 3 to 4 individuals for every person who agreed to participate requiring substantial time and effort. To make recruitment more efficient, we recommend more efforts on establishing credibility and assurances of confidentiality in the invitation email, and including a link to a study web page that describes the researchers and the purpose of the study.

This research has a few limitations. When screening, we did not ask about gender identity or sexual orientation, limiting our ability to purposively sample on these characteristics. Second, in our follow up survey, we did not ask about participants’ relative preferences for doing the interview by phone vs video chat. Phone interviewing affords many (but not all) of the benefits that video chat does. Finally, we recruited and conducted the interviews in English only. Thus, we cannot determine whether the same methods would have worked as well for adolescents who speak only other languages. However, recent US Census data indicates that most young people who speak a language other than English in the home report speaking English “very well” [32].

Overall, we agree with previous researchers [16] that online recruitment methods and video interviewing should be considered a viable first-choice option for researchers rather than as an alternative or secondary choice when face-to-face interviews cannot be achieved. In our experience the advantages outweigh the disadvantages. We hope these findings can contribute to a discussion of best practices in using online technologies for research in our field.

## Conclusions

The findings of this study have important implications for cognitive interviewers and qualitative researchers who focus on adolescent sexual and reproductive health, as well as other researchers who want to move towards research methods compatible with social distancing. We were able to combine multiple technologies for online recruitment and video interviewing to obtain a diverse sample of participants. Young people report feeling comfortable talking to researchers about sexual health and reproductive empowerment-related issues in their personal lives using a video meeting platform. Online recruiting and video meeting can contribute to increased diversity and inclusion of marginalized adolescents and young people in cognitive interviews for research. We encourage other researchers to use such methods to ensure that hard-to-reach populations are represented in research.

## Data Availability

All survey instruments and email templates are available from the corresponding author on reasonable request. The interviews were not recorded or transcribed to ensure participant confidentiality and privacy and thus are not available.

## Abbreviations

IRB: Institutional Review Board
UCSF: University of California, San Francisco
